# The Address of Dr. Hayden Coe

**Published:** 1861-06

**Authors:** 


*The Address of* Dr. Hayden Coe, *before the Medical Association of the State of Georgia, on yielding the Presidency to his successor, at the Annual Meeting in Atlanta, April* 1861.

Gentlemen: As I am about to leave the chair, to be occupied by one much more worthy than myself, I might well excuse myself from saying anything more, than to return you my hearty thanks for the honor which you have seen proper to bestow on me, especially when I consider how much has been said and written on the science of medicine, by those of much more ability than myself; nor will it be expected, that I shall say more than has been already said by others. I shall only endeavor to call your attention to some things which I may think most important for the improvement of our profession, by a retrospective view of the science of medicine.

Disease is the result of natural causes. It has its origin, most likely, from infringment upon nature's laws. Man is a creature of two natures, moral and physical, consequently, is under moral and physical law, neither of which can be violated without injury to those to whom they were given. The science of medicine has for its object, the cure of disease, and the preservation of health. This science consists in studying and knowing the laws of nature, as they are manifested in the human system in health and disease. It is true, of the origin of disease we know but little. Of the nature and treatment of disease among the Antideluvians, if any existed, we are entirely ignorant. Our knowledge of disease up to within a few centuries of the Christian Era, is very limited. The treatment of disease, for many centuries, was entrusted entirely to the care of the Priests, who believed in the celestial origin of disease; that it originated from Deity himself; that it was a manifestation of His displeasure, and that it could only be removed by some supernatural interferences; that something must be done to appease an offended Deity. Hence, their treatment consisted in little else than prayers and religious ceremonies. This was an error, which so fatally paralyzed every effort for improvement in the science of medicine, that century after century rolled around, until thousands of years had passed away without the science of medicine advancing a single step, or, was even known as a science. The error which they had imbibed, as to the origin of disease, so completely controlled them in the study and treatment, that it was impossible for it to advance, so long as it remained in their hands.

Medicine was hardly known as a science, until the days of Hippocrates, about four hundred and sixty years previous to the Christian Era. From that time to the present, he has been justly called the Father of Medicine. He wrested it from the hands of the priests and the trammels of superstition. He dared to combat the doctrine, which had been so long inculcated by the priests: the celestial origin of disease.

The first plan adopted by the ancients for the cure of disease, was laying the sick in public places, that those who passed by might see them and prescribe something, which they had seen used in cases which they supposed were similar, and was considered beneficial; and we not unfrequently have a similar kind of prescription made by those who visit our patients, even at the present day. It is said "that medicine was studied with some approach to system by the Egyptians;" but of their knowledge of medicine we know but little. Esculapius, a native of Thessolly, was the first individual who acquired any considerable reputation in the practice of medicine; but the extent of his knowledge of the human system, if he had any, is unknown to us. However, enough is known of Esculapius, to know he must have been a man of the first order of talent in his day, and his success in the treatment of disease might have appeared supernatural to the people in that dark age. Temples were erected to his honor, and he was worshiped as the God of Medicine. These temples were erected in different countries, both in Greece and Rome; the sick were carried to these temples to be healed. The priests who presided over these temples, were termed Asclepiadæ. The patients, who recovered, were required to record on tablets, a description of their disease and the remedy used for their recovery. By these means, a large number of facts were collected, from which, no doubt, some good resulted. One of these temples was erected at Cos, and had become, like some others, a kind of school of medicine, but was under the entire control of the priests. About this time, institutions were erected for gymnastic exercises. These also, were afterwards termed Gymnastic Schools of Medicine.

The Greeks at this time taking great interest in the study of Physical Science, had their minds prepared for the study of medicine. Pythagorus, a philosopher and a man of great learning, who preceded Hippocrates, was the first to disect animals and study comparative anatomy. He partially wrested the practice of medicine from the hands of the priests. Hippocrates, a native of Cos, his father a physician, a descendant of the Asclepiadæ, trained in the school at Cos, visited the Gymnastic School of Medicine, as well as several countries, in quest of knowledge, and commenced the study of medicine, by patient investigation, experience and observation. Hippocrates' chief merit consisted in his patient industry in watching, and his accuracy in recording the most minute circumstances occurring in disease, and from the facts thus collected, he, by induction, formed his general principles. Hippocrates never having disected the human body, knew little of Anatomy, and less of Physiology, consequently, his theories were based on the phenomena which occurred in disease. He being ignorant of Chemistry, Anatomy and Physiology, was not likely to avoid error in his theories of disease.

He believed in four elementary qualities: heat, cold, dryness and moisture ; and four cardinal humors: blood, bile, atrabile and phlegm, which harmonize very well with the theory of coction and cricis. He also believed in a living or vital principle, which presided over the system. He attributed disease to a derangement in these humors, or elementary qualities, in some way, by being in excess or vitiated. He thought this vital principle regulated and controlled these humors, and that fever was caused by an excitement produced by the effort of this vital principle to regulate these humors when deranged.

These theories reigned almost exclusively, from Hippocrates to Galen, and with some slight modification, for several hundred years after Galen. Plato and Aristottle were great philosophers among the ancients. Their means for obtaining knowledge, especially Aristottle's, were much better than Hippocrates'. But they were not willing to study medicine by the tedious process of experience and observation; they were unwilling to watch the phenomena as they occurred in disease. They adopted the method of commencing with general principles, and by a process of reasoning, to arrive at the particular phenomena as they occur in disease, a process which led them into error, as it has done many since their day. Aristottle added little or nothing to the theory of medicine, although he introduced many new facts in science.

In the study of medicine, we can adopt no general principles only such as are based upon well established facts. We cannot arrive at the truth in the science of medicine, by any process of reasoning from mental intuition ; our knowledge must come from observation.

At this epoch, three hundred years before the Christian Era, the great school at Alexandria, with its library of five hundred thousand volumes, was established. Here was the school of medicine. Men from various countries visited this institution, for the purpose of obtaining knowledge. A course of study in this institution was considered essential to prepare a man for the practice of medicine. The human body was first disected at this institution by Heirophilus and Erasistratus. They discovered the nerves, which, up to this time, had been confounded with the tendons and ligaments; and it would seem that they were unable to clearly distinguish between the tendons, ligaments and nerves. They discovered the use of the muscles, which, up to this time, were supposed to be passive, and merely coverings for the bones. A large collection of rare animals and plants had been collected at this institution, to assist in the study of natural history, which were of some service in the study of medicine. Disections of the human body did not continue long, not above two centuries, nor to any great extent; being prohibited by the domination of Rome. The Romans regarded the contact of corpses as profanation, although they could look upon the shedding of blood, not only on the battle field, but in their sports for public amusement, without any conscientious scruples. Consequently, the science of medicine had a backward tendency, while subtile discussions increased.

Not a single anatomist of any reputation ever had his origin in Rome; and although Rome increased in learning, and was styled the mistress of the world, yet she had few physicians of eminence. Celsus seems to be almost the only one, and there seems to be some doubt about his practicing medicine, yet, he was well acquainted with the science in his day, and practiced surgery pretty extensively, and was an eloquent and instructive writer.

At the school of Alexandria, there was more interest taken in discussing theories, than seeking after and teaching facts. They were divided into different sects, termed Dogmatists, Methodists, Empirics and Eclectics.

Dogmatism was taught in the school at Cos, and almost exclusively for several centuries anterior to the Alexandrian school. They attributed disease to certain internal causes, as the disturbance of the humors, or elementary qualities, heat, cold, dryness or moisture.

The Empirics were satisfied with giving a description of the symptoms as they occurred in each disease, without any attempt to search for the cause, or to a systematic arrangement. The Empirics destroyed their own doctrine for the want of a system, by the accumulation of facts with such slight shades of difference that they become a confused mass. In the science of medicine, we cannot do without theory; but our theories must be supported by well established facts.

The Methodists adopted a system after the system of Philosophy by Epecurus, which was very popular in that day. Supposing that the tissues, like all other bodies, were porus in health, atoms suited to these pores constantly passed through, and disease consisted in the interruption of these atoms

The Eclectics professed to follow neither sect, but to select the best from all; but they, like the Eclectics of the present day, have no system, nor do they select the best remedies from other systems, as they profess. If they did, they would tell us which they were, and give us their reasons for believing that their selection was the best.

We next have the illustrious Galen, who was born at Pergamas, educated in the school of that city, but traveled in several other countries in search of knowledge. Galen was a great admirer of Hippocrates, and he collected together many of his writings. It is not known to what sect Galen belonged. He disproved all, and might have called himself an Eclectic; but his writings, evidently, show that he was inclined to Dogmatism. He adopted the theory of four humors; also, he taught that there were three orders of Spirits—the natural, vital and animal. He thought the seat of the natural was in the liver, and presided over nutrition and growth; that the vital was seated in the heart, and dispensed warmth and life to every part of the body; and that the animal was seated in the brain, presiding over all the rest, communicated the power of motion and sensation to every part of the body through the medium of the nerves. It does not appear that Galen ever disected the human body, although he had considerable knowledge of anatomy, which he is said to have acquired from disecting monkies. Galen made some valuable improvements in Diagnosis and Prognosis.

From the death of Galen, at the close of the second century, for twelve hundred years, up to the revival of letters in Europe, in the fourteenth century, the science of medicine, like all other sciences, made little or no progress. Only two Greek writers of any originality, are spoken of after Galen. About the middle of the seventeenth century, the great Alexandrian library was destroyed by the Mahometans, from Arabia, and thus the literary and scientific sceptre passed from the hands of the Greeks and Romans into the hands of the Arabs. In several cities in Arabia, schools were established, one of which was at Cordovia, with a library of two hundred and twenty-four thousand volumes. Rhages, Avacenna, Hally Abas, and Albucasis, are the Arabian physicians who acquired most reputation. But they added nothing to the science of medicine—it even retrograded in their hands—except eruptive fevers, especially variola, which they were the first to describe and treat.

During this period of seven hundred years, only one Greek physician is mentioned—he resided at Constantinople, whose name was Actuarius. In Rome, the practice of medicine was again in the hands of the Clergy and Monks, with just about its former success. In Europe, Rogers, the founder of the Kingdom of Sicily, was the first to pass an ordinance, regulating the practice of medicine. Soon after, however, several other sovereigns passed similar ordinances, which, in the end, led to medical organization, by the institution of faculties and universities for degrees.

The first school was established at Salerno, where, after going through a course of study, from three to five years, the candidate was required to take an oath to be faithful to good behavior, and to submit to the rules of the profession; an oath, which should be required of every candidate at the present day.

About the beginning of the thirteenth century, the medical profession was declared by the Popes in Europe, to be incompatible with the sacerdotal office. This declaration was the first step towards a reformation in the science of medicine. At this time, almost the only schools in Europe for teaching medicine, were in the Cathedrals, and medicine was even sometimes practiced in these holy temples, but which must now be abondoned. But the Popes, in order to still hold control over the minds of the people, erected certain Episcopal schools into Universities, where Philosophy, Theology, Medicine and Law, were taught. Thus were created the great Universities throughout Europe, from which so many great men have eminated. We now find a few in this, (the thirteenth century,) who already begin to distinguish themselves in the science of medicine, among whom we may mention Villenuve, Lanfrane, Guy de Chanliue. With the fourteenth century, we commence the period of reformation in the science of medicine. There were some discoveries made about this time, which were important to science, and may have contributed something towards the rapid progress in the science of medicine. The invention of the compass, the invention of the microscope, but most of all, the discovery of the art of printing.

At this period, the literature of the Arabs was predominant; but the taking of Constantinople by Mahomet, seemed to be the mortal blow to Greek literature, but hastened its rise in the West. Several learned Greeks went to Italy, where they disseminated models of good literature. From thence, a taste for books, libraries, and sound learning, was disseminated throughout Europe. The ancient Greek and Latin works were now hunted up, and studied with great care, in those great Universities which had been previously established throughout Europe.

Linacre, who studied at the University of Oxford, in England, but afterwards visited the Universities in Italy, was the first Englishman to distinguish himself in the science of medicine. He caused the erection of two chairs, one at Oxford, the other at Cambridge, the object of which was to explain the works of Hippocrates and Galen. He also founded the College at London. At this time permission to practice medicine was only granted by the Bishops, consequently, it still was retained in the hands of Monks and the laity. Linacre, with his popularity with the Court of England, obtained letters patent, prohibiting any one from practicing medicine, who had not obtained a degree from one of the Universities of the Kingdom, and been examined by the

President of London College and three physicians, who were appointed for that purpose. Linacre was truly the restorer of the science of medicine in England.

From this time, the number of men who devoted their lives to the restoration of the science of medicine, are too numerous to be mentioned in detail. They first sought out the works of Greek writers, which were found to be far superior to the works of the Arabs. These works were translated, and studied. About this time, the beginning of the sixteenth century, the prejudice, which had existed from time immemorial, against disecting the human body, began to abate. The Popes, who were at the head of all scientific movements, withdrew their interdictions against disecting human bodies, and the Universities in Italy gave the first example of public disection of the human body. The result was, that in a few years, there were many distinguished anatomists.

No one, up to this time, had dared to differ on any point with Galen. Vesalius was the first who dared contradict the doctrines taught by Galen, many of which he refuted. Vesalius observed nature and her laws, more than the doctrines of Galen. Among those who followed the example of Vesalius, we find Eustacheus, Fallopius, Ferara, Pisa, Fabricius, and others. At this period, a chair of anatomy was established, and Amphitheatres erected. The scalpel took the place of the razor. The progress in anatomy was rapid, and many capital discoveries were made. The pulmonary circulation was now demonstrated, the valves in the veins discovered, and, although they came very near discovering the great circulation, yet the glory of that discovery was reserved for the immortal Harvey.

Surgery, during the middle ages, in the midst of the general decadence of science, fell yet lower than medicine, from the fact, that medicine was in the hands of the Priests, the only class of men who made any pretentions to literary attainments, while surgery was abandoned to ignorant butchers and barbers. A revolution in surgery commenced in the fourteenth century, increased during the fifteenth, and most of the anatomists whom we have mentioned in the sixteenth century were surgeons.

Medicine and surgery became again united, especially in France. Ambrose Pare, whose name is familiar to us, was the first to distinguish himself in the improvement and practice of surgery. His improvement in the treatment of gun-shot wounds, by omitting to apply the boiling oil, is well known, as well as his substituting the ligature for the cautery to arrest hemorrhage.

Obstetrics was not entirely neglected during the sixteenth century. It received the attention of several distinguished surgeons. Guilliman made some improvement in this branch. The Cæsarian operation was performed several times, an operation known to the ancients. We now have arrived at the period of the great reformation in medicine—the commencement of the seventeenth century. Several discoveries were made about this time in physical science, which might have stimulated those who were engaged in the study of medicine, to renewed exertions in their investigations. Gallileo calculated the laws of gravity, the weight of the atmosphere, the motion of the earth on its own axis, in its course around the sun. Gallileo abandoned the speculative theories of the ancients, and pursued the plan of observing the laws of nature, with patient industry. Kepler discovered with great exactness, the laws which govern the stars in their motion, and maintain them in harmony in their movements. Up to the seventeenth century, Physiology, as a science, was not known, nor could it be, for little, comparatively, was known of human anatomy until the sixteenth century, on which Physiology is founded. But, as we have already seen, anatomy was so extensively studied, and so minutely was every part described by the anatomists of the sixteenth century, that there was but little left for their successors of the seventeenth century to do in that branch. Consequently, they not only studied the parts, but the functions which they were destined to perform, which constitutes experimental Physiology. Up to this time, the liver was considered the origin of sanguification, the veins were considered the sole vessels for the blood, that it circulated by flux and reflux action, like the waves of the sea. The arteries were supposed to contain air or spirits. Galen thought the blood might be forced into arteries, but pass directly from the right to the left side of the heart. The ancients supposed that the extremities of the bronchia terminated in pulmonary veins, which they called arterial veins, because they supposed they conveyed the air to the heart, where it was drawn by the heat of the heart, which was considered the source of animal heat, for the purpose, as they supposed, of fabricating spirits, while the grosser parts of the air, which was not fit for fabricating spirits, was thrown out by the expiration.

I have related a few of these theories, which appear ridiculous to us, to show the importance of our being thoroughly acquainted with every organ in the system, and the functions which they are destined to perform, to avoid the most gross and ridiculous errors, as well as for the advancement of our science.

The discovery of the true circulation of the blood, by the immortal Harvey, in 1702, and published to the world in 1728, overturned the erroneous theories of the ancients.

"Theory," says Senac, "reduced to consequences, drawn from facts alone, is the light of practice." The same author wrote a work on the structure, action and diseases of the heart, in which it is said, that Diagnosis was carried to as great a perfection as it could be, until the discovery of percussion and auscultation. Malpighi demonstrated the progression of blood globules in the small vessels, and confirmed the reality of a communication between the arteries and veins. Several were engaged at this time in examining the movements of the chest, among whom was the great Haller. He established the fact, that the chest was enlarged during inspiration, by the contraction of certain muscles, and diminished during expiration by simple relaxation of the same. Also, that there never exists an empty space betweeen the lungs and sides of the chest, and lastly, that the air is not drawn into the lungs by the heat of the parts, as was supposed by the ancients, but by a certain law of gaseous bodies, to maintain equilibrium. Several pneumatic theories were now advanced, and then abandoned, until the close of the last century. Lavoirseir demonstrated the true theory of Respiration. This chemical theory was received by the world with great enthusiasm. It seemed to throw much light on two phenomena, Hematosis and Calorification, on which no satisfactory explanation had ever been given. But it was soon discovered that the lungs could not be the source of heat, as Lavoirseir supposed. The discovery of the lymphatic system was no less remarkable than the discovery of the circulation. Herophilus and Erasistratus saw the lacteals in the mesentery, but thought they were arteries filled with air. Galen thought the chyle was absorbed by the veins, one opinion, which prevailed to the middle of the seventeenth century, when some eight or ten contributed to establish the lymphatic system. The nervous systems were little known, and their functions not at all understood by Hippocrates or Galen, or any of the ancients. The anatomists of the sixteenth century described the nervous system more accurately, but knew little more of their functions. At the close of the last century, the splendid researches of Viussens, Haller, Mackel, Vic D Azyer, Scarpa and others, established beyond question, that the encephalon was the origin of sensation, motion, and the seat of the mind. Bichat divided the nerves into two systems: the cerebro-spinal and organic. Kepler, at the beginning of the seventeenth century, demonstrated the true theory of vision, which, until then, had been supposed to have its seat in the crystalline lens. Casserius, Duverny and Viussens demonstrated the function of hearing. Many conjectures and experiments have been made to explain the functions of the nervous system. Much has already been done, during the present century, in demonstrating the functions of the nerves, by Bell, Hall, and others. The result of their labor is so well known to you, it is unnecessary for me to say more on this subject. There is much still in relation to the functions of the nervous system, which we are unable to comprehend.

A multitude of hypotheses have been conceived to explain the mysterious function of generation, all of which may be resolved into two classes: Epigenesis and Evolution.

The first is, that a new being is formed in all its parts by an aggregation of molecules, over which a force presides regulating the particles. The second, the embryo preëxists in some form, is revified by fecundation, and commences a series of developements. The second class is again divided into ovarists and animalculists. In organic matter, the ancients admitted two kinds of properties; one they termed elementary, corresponding with our chemical, the other physical. But in living beings, from the days of Hippocrates, a force superior to those has been admitted. It has received different names, as vital, nature, numa, formative force, plastic force, &c., and the power which has been attributed to this force has varied more than the name.

The greatest philosophers and physiologists have agreed to regard each individual as endowed with an intrinsic force, which superintend the accomplishment of all its functions, unless interfered with by some material obstacle. Some physicians, in their management of disease, paid special attention to this force, and were called Hippocratists; others paid special attention to the condition of the humors—these were termed Humoralists; others regarded particularly the physical properties of the solids in particular porosity, attributing to the tisues only the quality of expansion and contraction; these were termed Methodists. The empirics disdain Physiology, and they had some reason for so doing in their day, for being ignorant of Anatomy they had no facts on which Physiology could be based. Therefore, it was made up entirely of hypotheses. If Physiology consisted merely in the study of vital force, as the ancients seemed to think, we might consider it less important. But Physiology has for its object, the careful investigation of the normal action of each and every organ in living beings, each function, the performance of which, requires three orders of forces; chemical, physical, and vital or formative force. About the middle of the seventeenth century, Glisson, professor at the University of Oxford, discovered or recognised a peculiar force in living tissue, which he termed *irritabillity*. Haller, by his immense and ingenious researches, elevated the hypothesis of Glisson to a demonstrated fact.

In 1757, Haller publishes his great Physiology. From this time Physiology has had a separate existence, independent of physics and chemistry. It was demonstrated that life has its laws, and its special forces, which must be studied after a particular method. Haller thought the tissues had a general and specific irritability. Fabre was the first to apply this doctrine of irritability to Pathology. He refuted the mechanical doctrine of Boerhave on inflammation. Bichat demonstrated that this force, irritability, exists in all the tissues, clearly tracing the characters which distinguish vital from physical forces. John Hunter clearly demonstrated that the blood, while it circulates in a living body, possesses properties which it loses when it issues from the vessel. Among those who contributed to the progress of Physiology during this period, are Winslow, Bernard, Sigeproy, Albinus, the Monroes, Douglass, Vic D Azyer, and others.

Those who have distinguished themselves, both in the practice and improvement of medicine, have become so numerous during the seventeenth and eighteenth centuries that a detailed account of each or any considerable portion of them would not be admissible on this occasion, nor is it possible to institute a comparison in the treatment of disease at the present day with the treatment even in the sixteenth century. The improvement is so manifest, that a mere glance at the subject is sufficient to demonstrate beyond a doubt that our science, the science of medicine, has kept pace with all physical science, in all its departments. Take Anatomy, Physiology, Pathology, Surgery, Obstetrics and Therapeutics, all, even the practical application of remedies. Of therapeutical agents that were used in the cure of disease previous to the days of Hippocrates, we know very little. Hippocrates used many active agents, such as blood-letting, emetics, cathartics, &c. At an early period in the history of medicine, we have seen a strong desire to discover the principle of life in living beings, and an equally strong desire to discover the essence of disease, the proximate principle—that they might attack this principle of disease with their therapeutical agent, and destroy it, or drive it out; and upon this idea was gotten up the first theory of the modus operandi of remedial agents. The idea was that the remedy must be opposed to the disease in order to drive it out, therefore, contrary, which established at once as they supposed, the doctrine of contraries, *contraria contrariis curantur.*

I believe there was no attempt at a classification of Materia Medica, until the days of Galen. It was then attempted by three individuals: Discaredes, Pliny and Galen. Each wrote a work on Materia Medica, but their classification was arbitrary and confused. The increase of remedial agents at this period, had made it necessary that an attempt at classification should be made. The therapeutics of Galen was used almost exclusively, with little alteration, up to the time of John Fernal, who wrote seven books on Therapeutics. He reduced to three genera all methods of medication, viz:

1st. To evacuate the excedent of the humors.

2nd. Purge or purify the humors.

3rd. Restore to the normal state of the parts which had been vitiated in their composition.

The mode in which he says these are effected, I deem unnecessary to relate. It has been said therapeutics is the capstone of the science of medicine. Anatomy, Physiology, Pathology, all have for their object, the better application of therapeutics in the cure of disease. The effects of all therapeutical agents on the system, either in a Physiological and Pathological condition, must be established by patient experience and observation. By no method of reasoning could we arrive at any conclusion as to the effect a single therapeutical agent would have until it is fully established by experience. Disease is abnormal action, produced by the effect of some agent, which we will term morbific. A remedy consists in nothing more than the use of some therapeutical agent, that will change the existing abnormal action, to one nearer normal, although it may be itself abnormal. This may be effected in one of two ways, or both: first, by removing or destroying the morbific agent which produced the disturbance, if it still exists; second, by modifying the abnormal action. The method by which the effect of a new therapeutical agent is known, is purely empirical; it is even often discovered by accident, but it requires a mind of genius to establish precisely what those effects are, the amount necessary to produce those effects, to free it from obnoxious qualities, and to arrange it in a proper class; and until this is done, it is of little use.

The practical application of therapeutics is not behind the age. Our diagnosis is much more certain. We administer our remedial agents with much more certainty. In the article quinine we have almost a specific, not only for periodical fevers, but for almost all periodical diseases. Our treatment of diagnosis in diseases of the eye, our diagnosis and treatment of diseases of the chest, also, diseases of the alimentary canal, uterine diseases, glandular diseases, diseases of the skin, diseases peculiar to women, the progress in the treatment of which has been rapid, and has not been excelled by any other science during the last or present century, to say nothing of the rapid improvement in obstetrics and in surgery. We can now carve the body with as little suffering to the patient as we could carve a turnip. In Hygiene, too, the prevention of varioloid by vaccination, has driven one of the greatest scourges with which the human family was ever afflcted, almost from the face of the earth. The system of drainage, which has been established at different places, the detection of contagious effluvia, the system of quarantine, as well as numerous instances of private hygiene, in regulating diet, exercise, change of climate, &c., are useful additions to science. Can it be said that our science does no good because we differ theoretically, which we have always done more than practically, and because we cannot always treat diseases as successfully as we desire?

If we have been led astray too often by subtile theories, let us examine the labors of those who preceded us, with a critic's eye, that we may be able to detect their errors, and escape them, and be the more careful not to commit any of our own. If it is difficult to distinguish between truth and error in the science of medicine, let us pursue the study with the more caution.

The science of medicine is an inductive science, and must be studied as such. General principles must be drawn from a collection of well established facts. Hippocrates commenced the study of medicine right—by experience and observation—but he failed to confine himself to the method of study with which he commenced; he not being satified with discribing the phenomena of disease as they occurred to him, sought by reason after the principle of life; he tried to ascertain in what it consisted, but could, like those who have followed him, only imagine a force or power, possessed by organic matter, not possessed by inorganic matter, which he termed vital, but attributed much greater powers to vital force, than has been attributed to it by modern physiologists. So with disease; he sought after the essence of some simple principle of disease, that he might attack it with some agent and drive it from the system, and as the result of his reasoning, he believed disease to be the disturbance of one of the four humors, or elementary qualities, which we have already named.

In the study of medicine our knowledge must be derived from sensation, and not from reasoning by mental intuition. Reason has only been given to us in the study of physical science, to guide experience, and the mind in attempting to pass the limits of sensation, mistakes its right and its power. By no process of reasoning from general principles, can we arrive at the particular phenomena, that will occur in any pathological condition of the system.

Galen adopted, as we have seen, most of the doctrines of Hippocrates, with some slight modification. The doctrines of Galen and Hippocrates were almost universally received and adopted by all, up to the sixteenth century; not that they were so perfect that there was no objection to them, for they were objected to by several, but they could offer nothing better in the place of them, nor was it possible for them to do so, for few disected the human body until the latter part of the sixteenth century. Therefore no facts could be collected through sensation, the only medium through which knowledge in the science of medicine can be obtained. But we see so soon as disecting the human body was commenced, by which a great many new facts were discovered, and collected together, from which general principles were drawn by induction, they were enabled to overthrow many of the doctrines of Galen, and almost completely revolutionized the science of medicine. They first examined every part of the human system, its structure, its organs. This was done in the sixteenth century. In the seventeenth, we see they were not satisfied with investigating the parts merely, but now, for the first time commenced investigating the function each part was destined to perform; to-wit: Physiology. This furnished a new supply of facts, from which general principles could be drawn by the same process of induction. And had they, in the seventeenth century not let their minds transcend the limits of what sensation furnished them, they would not have been led into error. But men are inclined to extremes, not being satisfied with describing the parts and the functions which had been made known to them through sensation, they, or at least many, commenced a course of reasoning by mental intuition. Besides they were also too exclusive. They were still like the ancients, not satisfied with describing the properties of organized matter, they wished to discover the principle of life, and the essence of disease—the proximate principle. This has led to many errors, both in Physiology and Pathology. The great Haller, when he discovered in living tissues the force which he termed irritability, thought he had discovered the vital force, or a force, by which he could account for all the phenomena in organized living beings, a force which was afterwards demonstrated by Bichat, to exist in all the tissues. This was an important discovery, one that will help us to account for many of the phenomena of living beings, but we cannot comprehend all the forces in living beings, yet, we must admit a force which is superior to the forces which govern inorganic matter. In living beings, we have three orders of forces: mechanical, chemical, and this vital or superior force. Strange as it may appear to us, yet we have theorists who have attempted to explain the action of all the functions, with each one of their forces exclusively. The chemical theorist sees nothing but chemical reaction and affinities in every action which takes place in the system. He adopts his treatment according to his theory, erroneous of course. The mechanical theorist sees nothing but mechanical force, obstruction, elasticity, pressure; explains everything on mechanical principles. His object in treating disease is to remove obstructions. Stahl comes in with his anamism, where disease is nothing more than the soul attacking the disease or morbific agent, to drive it out of the system. Fever is the reaction, or excitement, gotten up by these agents ; so he adopts the expectant mode of treatment, waiting for the soul to remove this intruder.

A more reasonable theory is one gotten up by Wm. Cullen, founded on the irritability of the tissues. Brown also founded a theory on the same principle, but much less plausible than Cullen's. He saw nothing but debility in the solids, consequently administered excitements for almost every disease. His theory was afterwards modified by Rosari, in which he divided diseases more equally into sthenic and asthenic. Brousais took an opposite view from Brown, and saw nothing but excessive irritation in every desease, therefore he adopted a sedative course of treatment. Next comes in Hahneman with his *similia similibus curantur* and infinitesimal doses. He must have concluded that the best method of curing disease was to let it alone and practice a deception on the mind. I think he might have gotten his idea of treatment from Stahl, a German who preceded him, whose theory we have already given.

Had all these theorists and their followers adhered strictly to their theories in their practice, the injury that they might have done the human family might have been immense.

But their practice, frequently, did not agree with their theory. When at the bedside of their patients, they laid aside their theories. But because some of these men of great minds have let their reason pass the limits for which it was given them, and have adopted physiological hypothesis for physiological truth, shall we say that pathological physiology is insufficient to furnish in part or whole a basis for therapeutical application? Shall we reject pathological physiology as a basis of our therapeutical application, as some have been inclined to do, and return to the simple primitive mode of pure empiricism? I say no, by no means. What did this simple method accomplish, for the science of medicine in over two thousand years; and what has pathological physiology done since 1757, the time when Haller wrote his great physiology? Let a history of the facts answer. Without a thorough knowledge of Anatomy, we cannot comprehend physiology, and a thorough knowledge of physiology, is essential that we may understand pathology and on our knowledge of pathology, will depend the correctness of our Diagnosis, and on the correctness of our Diagnosis, will depend the success of our remedial applications. It is true that the method by which the first effect of any new therapeutical agent is known is purely empirical, and is often discovered by accident, as we have already stated. But after its effects have been determined by patient experience and observation, our Diagnosis will and ought to influence us in the selection of our therapeutical applications. I cannot omit refering to another method of studying our science, which has received special attention during the present century, as well as some attention during the past; a method from which much practical knowledge has been derived. I mean clinical instruction. There we see our science practically demonstrated. This important mode of study should never be neglected. It is not only necessary that we be thoroughly acquainted with every part of the human system, and its functions, both in a physiological, and pathological condition, and thereby form a correct Diagnosis, but it is equally as essential that we be as thoroughly acquainted with the properties of every therapeutical agent, and their effect on the system both in a physiological and pathological condition, which has been determined by long experience and observation. I do not mean by this that we should use no agent whose effects have not been so determined; but new agents should be used cautiously, and we should call to our aid all collateral science of which a knowledge of chemistry is essential. Yes, chemistry is all important—it is part of the science of medicine itself.

Now, if what I have said is true, is it not important for us to be more diligent in the study of the labors of those who have preceded us, that we may profit by the great truths which they have established, and escape the errors which they have committed? Shall the generation who shall succeed us, have it to say that we have added nothing to our science, or worse, attempted to establish errors? Is there nothing for us to do? Have those who have preceded us accomplished all? Let those who are engaged both in teaching and practicing medicine ponder this question well. Let us feel the responsibility that rests upon us? Let us realize the importance of our profession to humanity, although, they may not appreciate it as much as we think they ought. Let us first respect it ourselves, and maintain the dignity which it deserves, and I am clearly of the opinion that it will be respected by the people of this enlightened age. I am well aware that there are many difficulties in the way to hinder the progress of our science, which I might refer to had I not already detained you so long, but there are none so formidable but what they can be overcome by a reasonable exertion on our part. Never has the profession, in my opinion, been placed under more favorable circumstances for improvement, than at the present time.

Climate, variety of soil, government—all are said to have their influence on the progress of science, a fact clearly demonstrated by history. We, with a climate unsurpassed, a soil with a greater variety of production than any country in the world, a Government which is said by better judges than myself, to be an improvement upon the best Government the world ever saw. With these and other advantages which if time permitted, I might enumerate, are we not able to overcome any and all the difficulties which surround us, and not only maintain the honor and dignity of our profession, but elevate it to a height that has never been surpassed by any age or country? Let us now, both teacher and practitioner, resolve to renew our exertions for the improvement of our science, and I hope that there will be some step taken before the close of this meeting, to call a Southern Convention, and when we shall come together, we can say the same that our politicians now say, that we, for once, are all of one mind. And now before I close, I must offer you my apology for having detained you so long, and return you my sincere thanks, for the respect shown me by the members of this association.

